# Transplantation of clinical-grade human neural stem cells reduces neuroinflammation, prolongs survival and delays disease progression in the SOD1 rats

**DOI:** 10.1038/s41419-019-1582-5

**Published:** 2019-04-25

**Authors:** Cristina Zalfa, Laura Rota Nodari, Elena Vacchi, Maurizio Gelati, Daniela Profico, Marina Boido, Elena Binda, Lidia De Filippis, Massimiliano Copetti, Valentina Garlatti, Paola Daniele, Jessica Rosati, Alessandro De Luca, Francesca Pinos, Laura Cajola, Alberto Visioli, Letizia Mazzini, Alessandro Vercelli, Maria Svelto, Angelo Luigi Vescovi, Daniela Ferrari

**Affiliations:** 1 Department of Biotechnology and Biosciences, University of Milano-Bicocca, Piazza della Scienza, 2, 20126 Milan, Italy; 2 Fondazione IRCCS Casa Sollievo della Sofferenza, Production Unit of Advanced Therapies (UPTA), Institute for Stem-Cell Biology, Regenerative Medicine and Innovative Therapies (ISBReMIT), 71013 San Giovanni Rotondo, Foggia, Italy; 3 Neuroscience Institute Cavalieri Ottolenghi, Department of Neuroscience “Rita Levi Montalcini”, University of Torino, Torino, Italy; 4 Fondazione IRCCS Casa Sollievo della Sofferenza, Cancer Stem Cells Unit, Institute for Stem-Cell Biology, Regenerative Medicine and Innovative Therapies (ISBReMIT), 71013 San Giovanni Rotondo, (FG) Italy; 5 Fondazione IRCCS Casa Sollievo della Sofferenza, Regenerative Medicine and Innovative Therapies (ISBReMIT), 71013 San Giovanni Rotondo, (FG) Italy; 6 Fondazione IRCCS Casa Sollievo della Sofferenza, Bioinformatics Unit, Viale dei Cappuccini, 71013 San Giovanni Rotondo, (FG) Italy; 7 Fondazione IRCCS Casa Sollievo della Sofferenza, Molecular Genetics Unit, Viale dei Cappuccini, 71013 San Giovanni Rotondo, (FG) Italy; 8 Fondazione IRCCS Casa Sollievo della Sofferenza, Cellular Reprogramming Unit, San Giovanni Rotondo, (FG) Italy; 9 StemGen S.p.a, Milan, Italy; 10 Centro Regionale Esperto SLA Azienda Ospedaliero-Universitaria “Maggiore della Carità”, Novara, Italy; 11 Department of Bioscience, Biotechnology and Biopharmaceutics, University of Bari Aldo Moro, Bari, Italy

**Keywords:** Amyotrophic lateral sclerosis, Neural stem cells

## Abstract

Stem cells are emerging as a therapeutic option for incurable diseases, such as Amyotrophic Lateral Sclerosis (ALS). However, critical issues are related to their origin as well as to the need to deepen our knowledge of the therapeutic actions exerted by these cells. Here, we investigate the therapeutic potential of clinical-grade human neural stem cells (hNSCs) that have been successfully used in a recently concluded phase I clinical trial for ALS patients (NCT01640067). The hNSCs were transplanted bilaterally into the anterior horns of the lumbar spinal cord (four grafts each, segments L3–L4) of superoxide dismutase 1 G93A transgenic rats (SOD1 rats) at the symptomatic stage. Controls included untreated SOD1 rats (CTRL) and those treated with HBSS (HBSS). Motor symptoms and histological hallmarks of the disease were evaluated at three progressive time points: 15 and 40 days after transplant (DAT), and end stage. Animals were treated by transient immunosuppression (for 15 days, starting at time of transplantation). Under these conditions, hNSCs integrated extensively within the cord, differentiated into neural phenotypes and migrated rostro-caudally, up to 3.77 ± 0.63 cm from the injection site. The transplanted cells delayed decreases in body weight and deterioration of motor performance in the SOD1 rats. At 40DAT, the anterior horns at L3–L4 revealed a higher density of motoneurons and fewer activated astroglial and microglial cells. Accordingly, the overall survival of transplanted rats was significantly enhanced with no rejection of hNSCs observed. We demonstrated that the beneficial effects observed after stem cell transplantation arises from multiple events that counteract several aspects of the disease, a crucial feature for multifactorial diseases, such as ALS. The combination of therapeutic approaches that target different pathogenic mechanisms of the disorder, including pharmacology, molecular therapy and cell transplantation, will increase the chances of a clinically successful therapy for ALS.

## Introduction

Amyotrophic lateral sclerosis (ALS) is a progressive disease that targets primarily motor neurons (MNs) and leads to fatal paralysis^1,2^. The molecular mechanisms that initiate and drive the inherent neurodegenerative process are largely unknown. Recent reports strongly implicate neuroinflammatory processes, such as astrogliosis, microgliosis and the infiltration of T lymphocytes, in the progressive degeneration of MNs:^3–5^ These non-cell-autonomous mechanisms are promising therapeutic targets.

ALS is a multifactorial disorder that involves genetic as well as environmental factors. Most (90–95%) of ALS forms are sporadic (sALS), while about 10% of cases are familial (fALS) and are associated with mutations in specific genes, such as superoxide dismutase 1 (*SOD1*, 20% of fALS), *TARDBP* and *FUS* (5% of fALS) and *C9ORF72* (40% of fALS)^6–8^. SOD1 was the first mutated protein that was correlated with the development of ALS^9^, and it has been leveraged to generate animal models of ALS—these include the SOD1 rats used here^10^, which reproduce many of pathological and symptomatic features of the human disorder and have been used for developing therapeutic strategies, such as stem-cell transplantation.

Preclinical studies show that intraspinally transplanted human neural stem cells (hNSCs) provide trophic support to damaged cells, and also modulate the immune cell environment, thus acting on disease mechanisms at multiple levels;^11–20^ based on these results, the approach was translated into the clinic, and two phase I^21–23^ and phase II^24,25^ studies with use of hNSCs have been successfully completed. The exact mechanisms through which these cells exert their beneficial effects have not been completely identified. Moreover, the use of hNSCs derived from different CNS sources, using a variety of methods, further confounds the direct comparisons of findings from different labs. For clinical applications, a standardised protocol that guarantees the reproducibility, safety and efficacy of hNSCs is of utmost importance. Our group has established a Cell Factory and Biobank at the Hospital Santa Maria in Terni that is currently producing hNSC lines from the foetal brain, using methods^26^ that are fully compliant with current Good Manufacturing Practice (cGMP) guidelines, and are approved for clinical applications by the Italian Medicine Agency (AIFA, aM 154/2018). The cell lines are characterised by a consolidated paradigm to assess their stemness and safety. Consistent with this rigorous approach, the hNSCs have been successfully used in the phase I trial for ALS patients^23^, EudraCT 2009–014484–39 NCT01640067), and are also currently being evaluated in a phase I study for the treatment of Secondary Progressive Multiple Sclerosis (EudraCT 2015–004855–37 NCT03282760).

As a complement to the phase I trial, and preliminary to phase II, we evaluate here the therapeutic potential of using a GMP-grade hNSC line in the SOD1 rat model of ALS. hNSCs were delivered by intraspinal cord transplantation, using the same strategy as for ALS patients^23,24^. Because we intended to unveil the role played by hNSCs in delaying neural degeneration, e.g., by modulating neuroinflammation^11^, we also evaluated the symptomatic hallmarks of ALS, together with astrogliosis and microgliosis, at different stages of disease progression

## Results

### Hallmarks of symptomatic progression in SOD1 rats

**Fig. 1:**
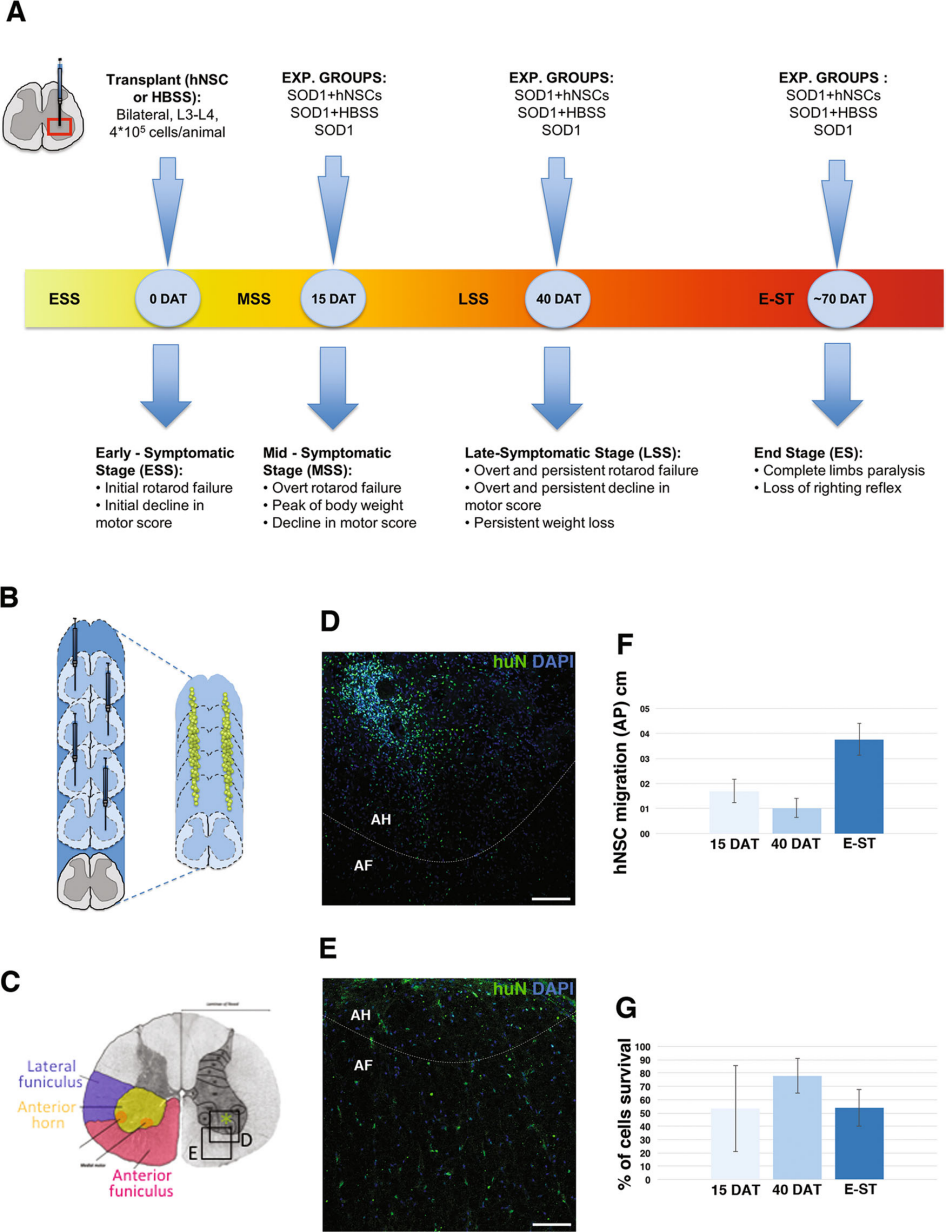
Experimental plan and migration of transplanted hNSCs. **a** Experimental plan: rats were transplanted with hNSCs or HBSS when showed the initial symptoms of the disease (ESS), age-matched not-transplated controls were also analysed. Behavioural test was performed for all the durations of animals life. Histological analysis of the spinal cord was performed at 15DAT, 40DAT and at E-ST, that approximately correspond to MSS, LSS and E-ST. **b** Schematic showing the transplant strategy and migration of hNSCs (green dots). **c** Schematic representation of the spinal cord organisation. hNSC or HBSS were transplanted into the anterior horns (asterisk) and were able to radially migrate into the anterior funiculus (red) and the lateral funiculus (blue). **d** Injection site with an evident cluster of grafted hNSCs (huN +) in the anterior horn of an animal at 40DAT. **e** At E-ST hNSCs (huN +) reached a higher grade of dispersion into the parenchyma. **f, g** Charts showing the differences in hNSCs migration (**f**) and survival (**g**) at 15, 40DAT or E-ST. ESS early-symptomatic stage, MSS mid-symptomatic stage, E-ST end stage, DAT days after transplantation, a.f. anterior funiculus, a.h. anterior horn. Scale bars: 100 μm

We evaluated disease progression in SOD1 rats by monitoring the gradual deterioration of the motor system as reflected by rotarod performance, motor score and weight assessment, in *n* = 22 SOD1 rats (CTRL) compared with *n* = 9 wild-type (WT) animals^27–29^. This analysis led us to identify the following four subsequent clinical stages (Fig. 1a), based on the worsening of symptoms:

(i) *Early-symptomatic stage (ESS)*: this phase started with subtle decrease in limb control (resulting in a 5% reduction in the motor score) and/or a two consecutive failures of the rotarod test in the same week. These changes occurred variably between P92 and P115 (Fig. 1).
(ii) *Mid-symptomatic stage (MSS)*: the symptoms became gradually more evident during the following month (~27 days after ESS; Fig. 1a), at the end of which a decline in the weight of SOD1 rats, due to denervation-induced muscle atrophy, started to become evident, (peak body weight at 21 days after ESS, orange arrow in Supplementary Fig. 1A). Concomitantly, motor impairments were now overt and stable, with a significant decrease in the motor and rotarod performance scores relative to the scores recorded for ESS (18%, *p* < 0.05 vs. WT rats, orange arrow in Supplementary Fig. 1B; and 25%, *p* < 0.01 vs. WT, orange arrow in Supplementary Fig. 1C, respectively).
(iii) *Late-symptomatic stage (LSS)*: transition from MSS to LSS was characterised by a rapid decline of weight and motor functions (Fig. 1a). At 40 days after ESS, the body mass was reduced by 10% (red arrows in Supplementary Fig. 1A) and the performance on both motor tests was decreased by 50% (red arrows in Supplementary Fig. 1B, C). At about 60 days after ESS, SOD1 rats displayed minimal motility and were unable to perform the rotarod challenge (Supplementary Fig. 1A–C).
(iv) *End stage (E-ST*): the day of E-ST was established by the loss of the righting reflex (∼70 days after ESS, Fig. 1a).

The day of transplantation was established as that day when early motor symptoms first appear (referred to as day 0, occurring within the ESS, at ~P105-P115; Fig. 1a and yellow arrows in Supplementary Fig. 1A–C). SOD1 rats received four bilateral injections of hNSCs (hNSC group, a total of 400,000 cells/animal, *n* = 15), or of only HBSS (HBSS group, *n* = 15) into the anterior horn of the lumbar spinal cord (L3–L4; Fig. 1b, c). Another control group of SOD1 rats did not receive any surgical procedure (CTRL group, *n* = 22).

Histological analysis of the spinal cord was performed at three different time points (Fig. 1a): 15 days after transplantation (15DAT) during the MSS, 40DAT during the LSS and at E-ST.

### Transplanted hNSCs survive, migrate and differentiate in the spinal cord of SOD1 rats

Viable hNSCs grafts (huN +, Fig. 1d–e) were found in all animals. At 15 and 40DAT, most of hNSCs were integrated into laminae VIII–IX of the anterior horns (Fig. 1c, d, AH) and few had migrated into the lateral and anterior funiculi (Fig. 1d, AF). At E-ST, huN + cells were dispersed within the spinal cord and occurred individually (not clustered, Fig. 1e); the cells had migrated extensively along the rostro-caudal axis of the cord, from the thoracic and sacral cord segments (1.69 ± 0.47 cm at 15DAT, 1.02 ± 0.39 cm at 40DAT and 3.77 ± 0.63 cm at E-ST, Fig. 1f).

**Fig. 2:**
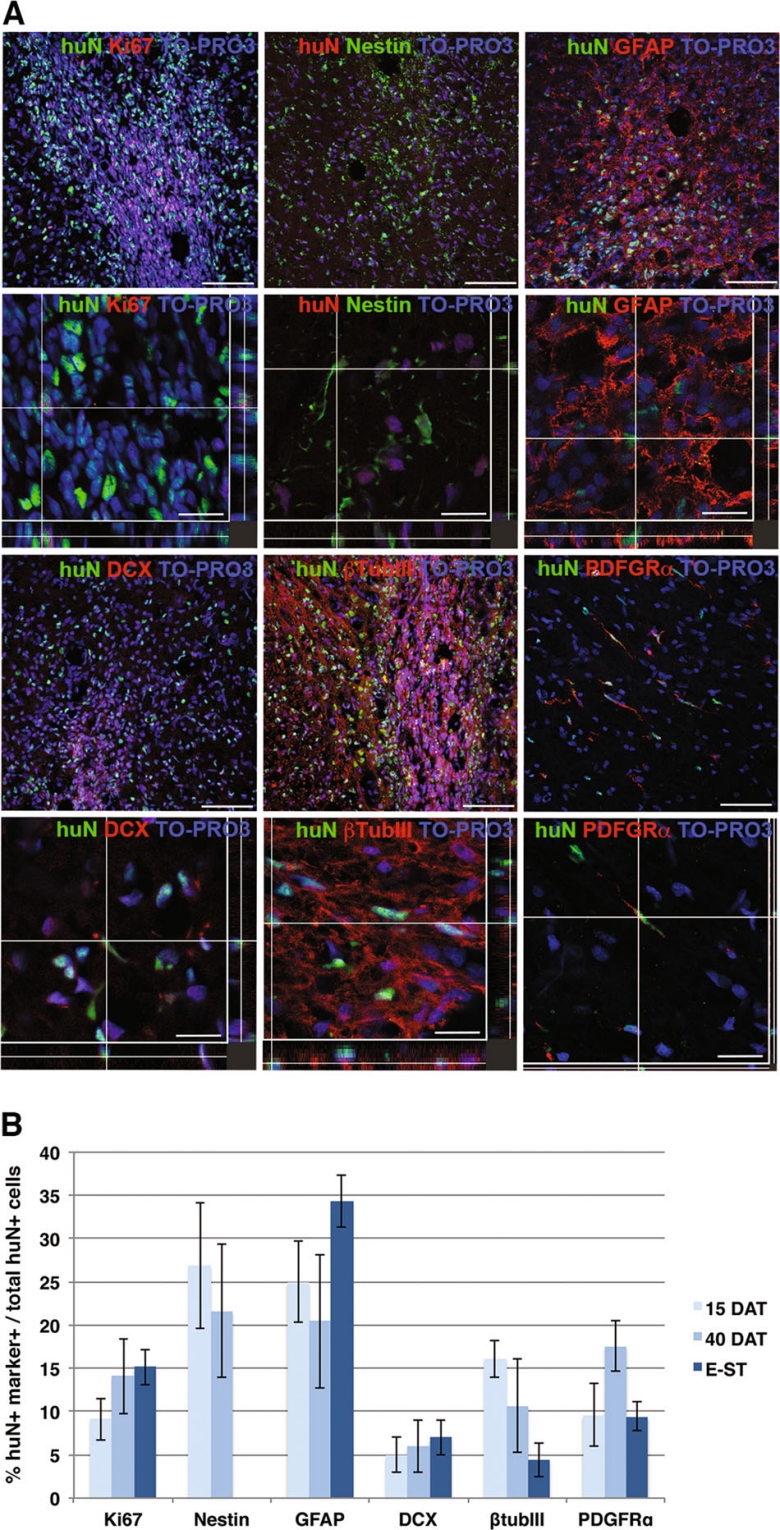
Differentiation of transplanted hNSCs within the SOD1 rat spinal cord. **a** Representative confocal images showing the colocalization of the human nuclear antigen (huN) with proliferating cells (Ki67) and neural phenotype markers (Nestin, GFAP, DCX, btubIII and PDGFR-a) in transplanted hNSCs at 40DAT. **b** Quantification of each markers. Scale bars: 75 μm for the maximum projection images and 15–25 μm for the colocalization images

A high percent of the transplanted cells had survived, and were detected at all three time points of analysis (53.29 ± 32.34% at 15DAT; 77.81 ± 12.89% at 40DAT; and 53.65 ± 19.41% at E-ST—Fig. 1g, *n* = 3/time point). At each time point, we also detected a similar fraction of transplanted cells that were actively proliferating (Ki67 + cells: 9.11 ± 2.5% at 15DAT, 14.1 ± 4.3% at 40DAT and 15.2 ± 2.1% at E-ST; Fig. 2a, b), suggesting that new progenitors were generated in vivo. Transplanted hNSCs (Fig. 2a, b) expressed Nestin (26.9 ± 7.3% at 15DAT; 21.7 ± 7.7% at 40DAT; and sporadic at E-ST) and differentiated into astroglia (GFAP +: 25 ± 4.7% at 15DAT, 20.5 ± 7.7% at 40DAT and 34.4 ± 3% at E-ST), neurons (β-Tub III +, 16.1 ± 2.2% at 15DAT, 10.7 ± 5.4% at 40DAT and 4.5 ± 2% at E-ST) and oligodendrocyte precursors (PDGFRa +: 9.6 ± 3.7% at 15DAT; 17.6 ± 2.9% at 40DAT; and 9.4 ± 1.6% at E-ST). These data indicate that hNSCs efficiently survive, transiently proliferate and differentiate in the spinal cord of SOD1 rats.

### Transplanted hNSCs prolong survival and delay the progression of motor symptoms

**Fig. 3:**
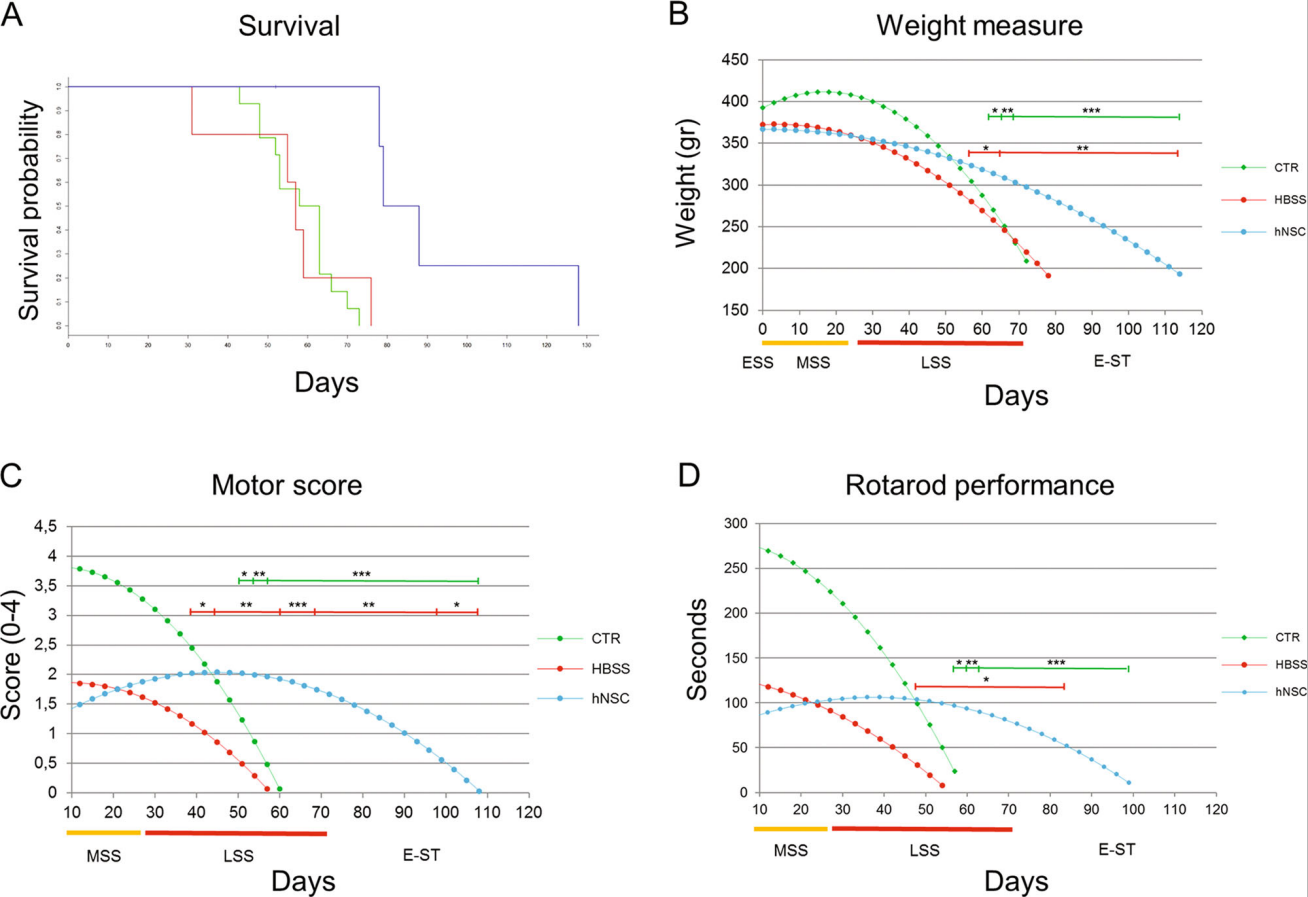
Extention of lifespan and slow-down of behavioral deterioration in hNSCs-treated rats. **a** Kaplan–Meier survival probability analysis, *n* = 14 for untreated SOD1 (CTRL, green) and *n* = 5 for HBSS-treated SOD1 (HBSS, red) and hNSCs-treated SOD1 (hNSCs, blue). **b–d** Trend of weight, motor score and rotarod performance decline in CTRL (green, *n* = 22), HBSS (red, *n* = 15) and hNSCs (blue, *n* = 15) rats. The curves were obtained by appliyng statistical modelling of the progressive reduction of the mean weight value (**b**) or mean motor score value (**c**) or the mean time spent on the rotarod (**d**). Where the hNSCs-treated animals showed a statistically significant improved value respect to CTRL is indicated with asterisks above the green line, respect to HBSS animals with asterisks above the red line. On the *x* axis, the days are shown after ESS and coloured bars indicate the stage of the disease. *p ≤ 0,05; **p ≤ 0,01; ***p ≤ 0,001. Data are reported as mean ± SEM

The median survival time for hNSCs-transplanted rats was 83.5 days, ~23 days longer than the control groups, which had median survival times of 60.5 days (CTRL animals, HR: 0.08; 95% CI = 0.01–0.65; *p* = 0.018) and 57.0 days (HBSS group, HR: 0.08; 95%CI = 0.01–0.72; *p* = 0.024, see Fig. 3a). The curves for the two control groups were overlapping (HR: 0.94; 95% CI = 0.31–2.79; *p* = 0.905).

Disease progression was significantly delayed in hNSCs-transplanted animals, relative to CTRL and HBSS-treated animals (Fig. 3b–d), as evaluated by weight decline (*p* < 0.0001 versus CTRL and *p* = 0.023 versus HBSS), motor score (*p* < 0.0001 versus CTRL and *p* = 0.001 versus HBSS) as well as rotarod performance (*p* < 0.0001 versus CTRL and *p* = 0.011 versus HBSS). The transplantation surgery led to a worsening of motor performance for about 10 days, during which time the treated rats were not always able to perform on the behavioural tests. In fact, during the MSS, the performance of both treated groups (hNSC and HBSS) was similar (*p* > 0.5), and was significantly worse than that of the CTRL group (*p* < 0.03).

At the LSS stage, the performance of the hNSCs-treated rats appeared to be stabilised, and was reliably better than that of the HBSS-treated animals. The motor score was 2.02 ± 0.3 for hNSC and 1.17 ± 0.31 for HBSS at 39DAT (*p* < 0.05, Fig. 3c); the rotarod score was 103.37 ± 22.84 s for hNSC and 30.47 ± 25.99 s for HBSS at 48DAT (*p* < 0.04, Fig. 3d); and the weight was 323.51 ± 12.56 g for hNSC rats and 280.12 ± 15.86 g for HBSS rats at 57DAT (*p* < 0.04, Fig. 3b). Of note, disease progression was ameliorated in hNSCs-transplanted animals, even when compared with CTRL animals, although this difference was evident later than relative to the HBSS groups. In particular, the motor scores were 2.02 ± 0.31 for hNSC rats and 1.23 ± 0.25 for CTRL at 51DAT (*p* < 0.05, Fig. 3c); the rotarod score was 96.62 ± 24.13 s for hNSC rats and 23.65 ± 20.94 s for the CTRL group at 57DAT (*p* < 0.03, Fig. 3d); and weights were 313.89 ± 13.04 g for hNSC rats versus 269.96 ± 12.59 g for the CTRL group at 63DAT (*p* < 0.02, Fig. 3b).

At E-ST (60DAT), while animals in both control groups had almost completely lost their motor ability (rotarod and motor score values = 0), motor function was still partially preserved in the hNSCs-transplanted animals (50% of motor score, 1.93 ± 0.34, Fig. 3c), as was their ability to run on the rod (30% of rotarod performance, 93.47 ± 24.54 s, Fig. 3d).

### hNSCs induce a significant decrease in the degeneration of MNs

**Fig. 4:**
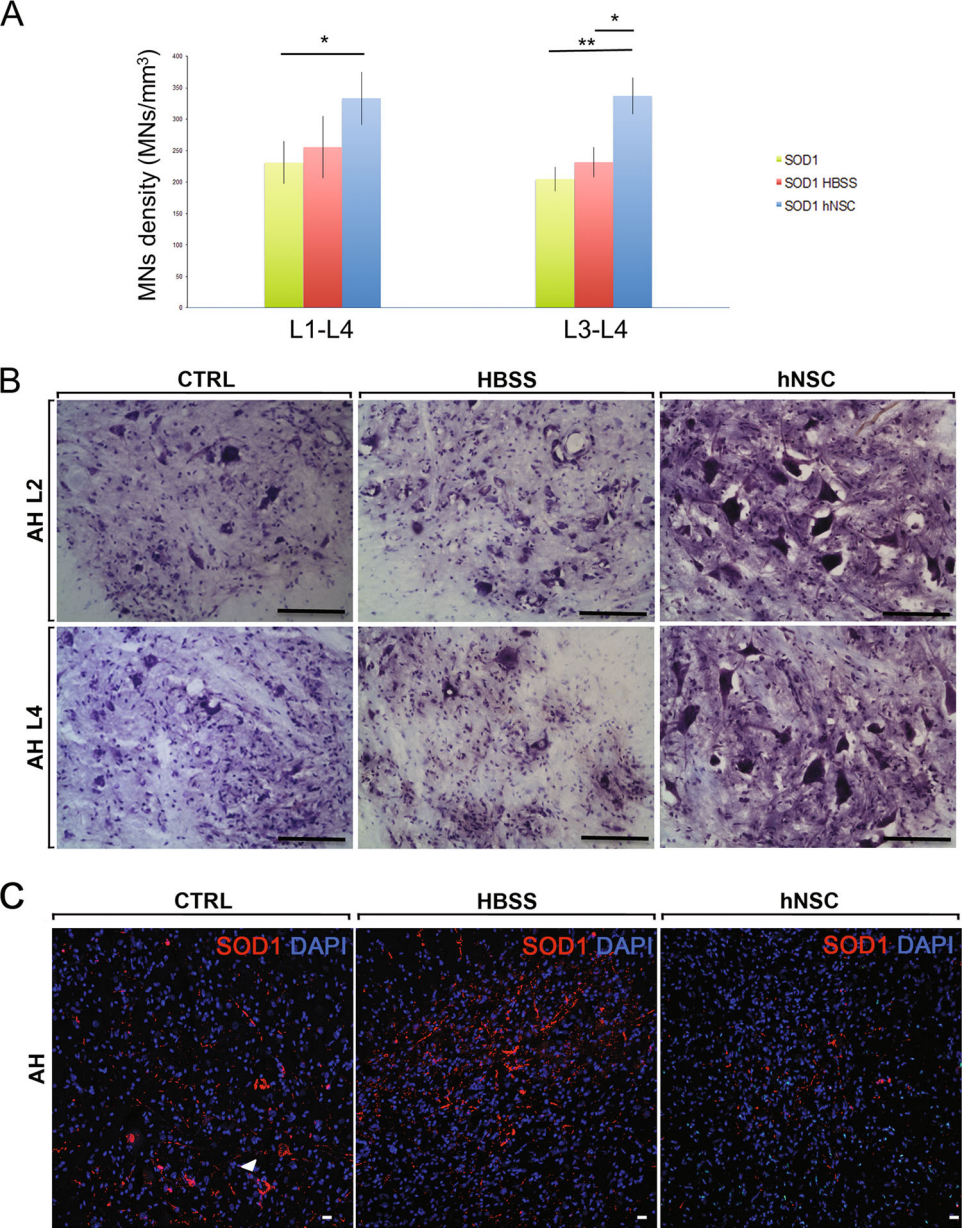
Reduction of motor neurons degeneration and accumulation of misfolded SOD1 in hNSCs-treated SOD1 rats. **a** Chart showing the motor neurons density (MNs/cm^3^) in the L1-L4 tract, *n* = 5 for each experimental group. *p ≤ 0.05; **p ≤ 0.01. Data are reported as mean ± SEM. **b** Representative images of the MNs in the anterior horn (AH) of the spinal cord (Nissle staining). Scale bars: 100 μm. **c** Representative confocal images of misfolded SOD1 deposits in the AH of CTRL, HBSS or hNSCs rats (*n* = 3/group) at 40DAT. Scale bars: 20 μm

The functional data were validated by histopathological findings, which mirrored a significant delay in the degeneration process of MNs at 40DAT, as demonstrated by the results of stereological analysis of MN cell density in the treated region of the spinal cord (Fig. 4). Preservation of MNs was maximal around the cell injection site, namely within segments L3–L4: hNSCs-treated SOD1 rats displayed a significantly higher density of MNs (336.65 ± 28.73 MNs/mm^3^) relative to control groups (204.25 ± 19.47 for CTRL *p* ≤ 0.01 and 231.60 ± 24.05 for HBSS *p* ≤ 0.05; Fig. 4a, b). To corroborate the beneficial effect of hNSCs, we evaluated the presence of misfolded SOD1 deposits by using the SEDI antibody^30,31^. Of note, in the anterior horns of hNSC animals, at 40DAT, SOD1 deposits appeared reduced respect to both control groups (Fig. 4c), in which SOD1 was apparent and predominantly accumulated within MNs (Fig. 4c, arrow).

### hNSCs immunomodulate reactive astrogliosis and microgliosis

**Fig. 5:**
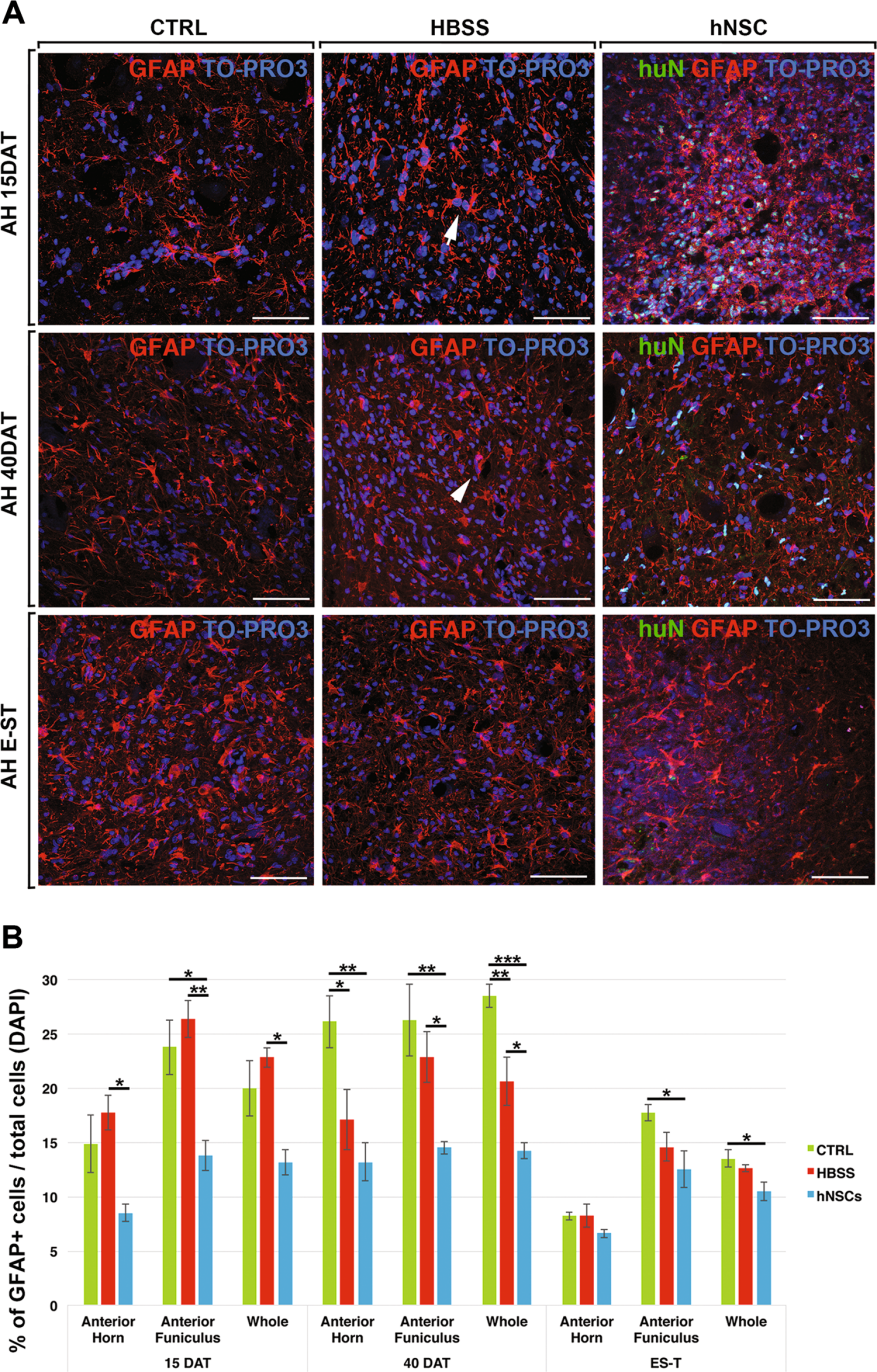
Reduction of astrogliosis in hNSCs-treated rats. **a** Representative confocal images of activated astroglial cells (GFAP+) in the anterior horn (AH) of CTRL, HBSS or hNSCs rats. **b** Chart showing the quantification analysis of the number of GFAP+ cells in the anterior horn, anterior funiculus or whole (anterior horn, anterior funiculus and lateral funiculus). Scale bars: 75 μm

**Fig. 6:**
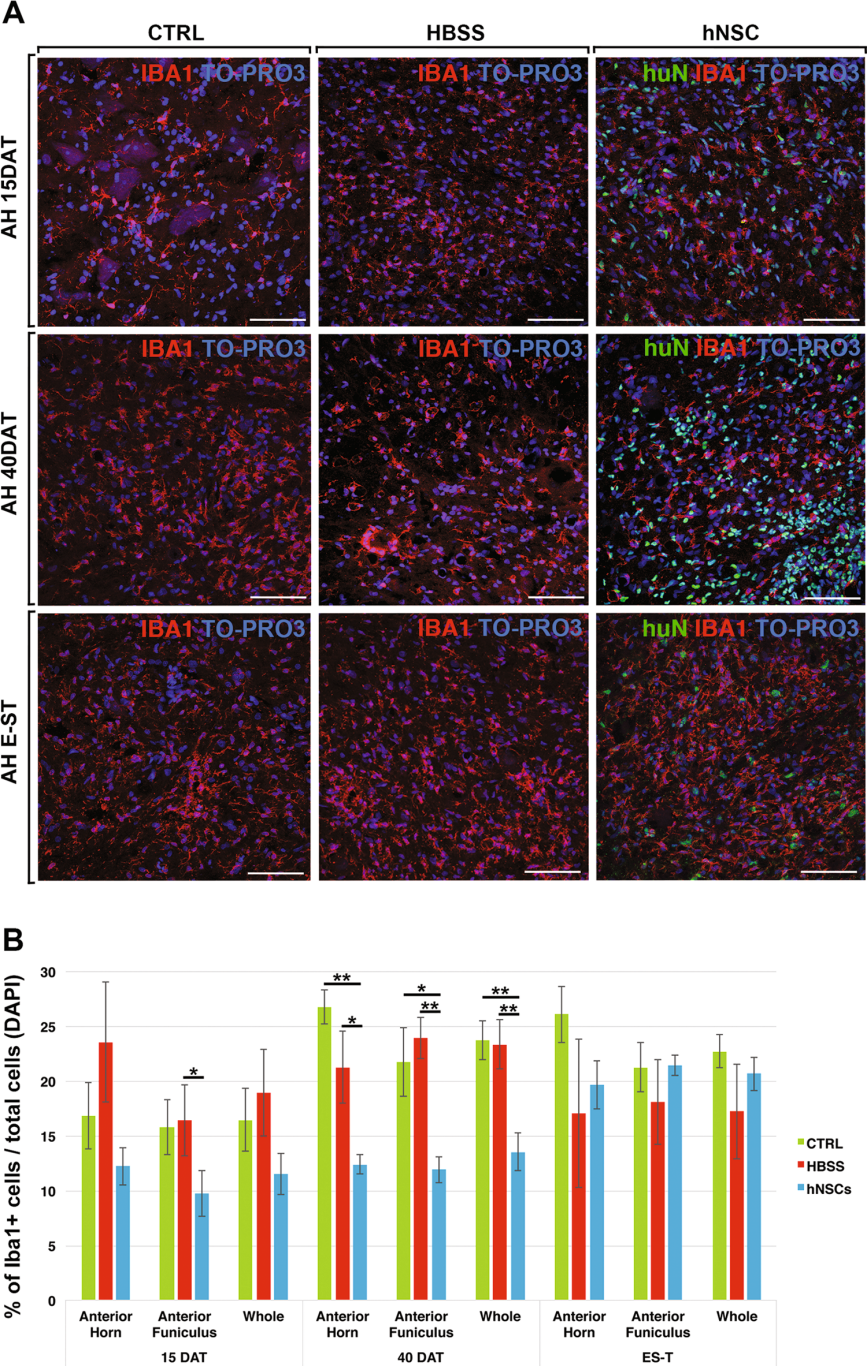
Reduction of microgliosis in hNSCs-treated rats. **a** Representative confocal images of activated microglial cells (Iba1+) in the anterior horn (AH) of CTRL, HBSS or hNSCs rats. **b** Chart showing the quantification analysis of the number of Iba1+ cells in the anterior horn, anterior funiculus or whole (anterior horn, anterior funiculus and lateral funiculus). Scale bars: 75 μm

**Fig. 7:**
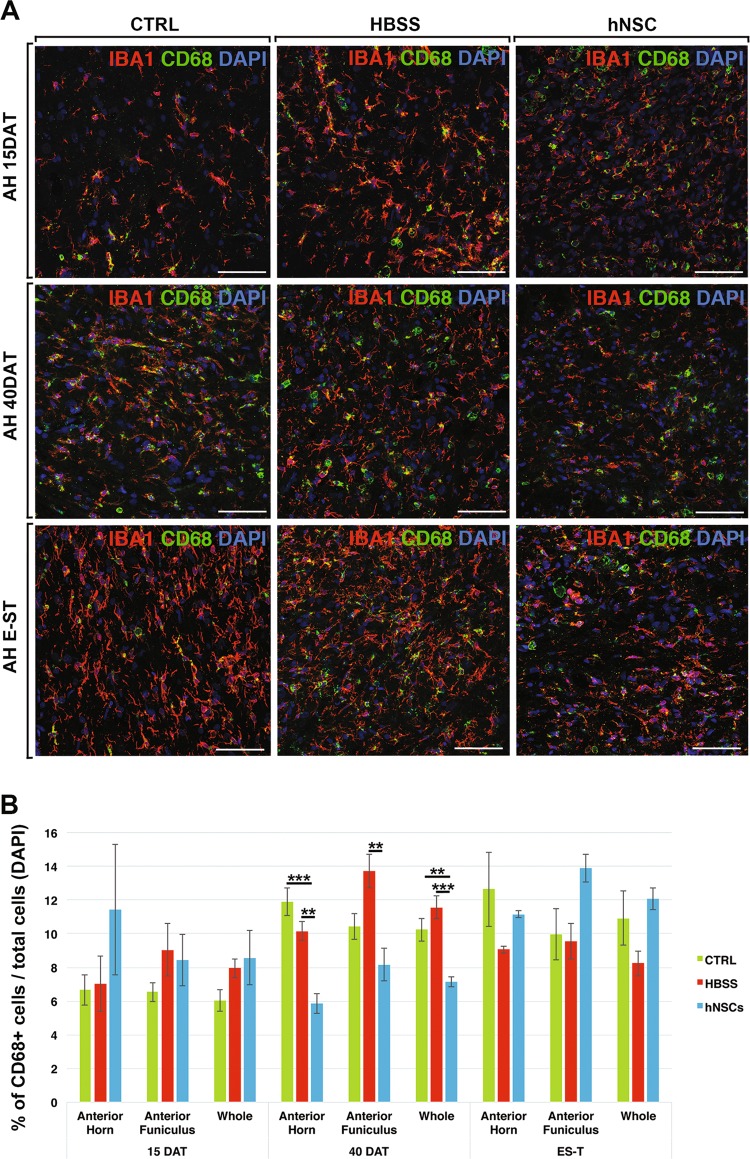
Reduction of activated CD68+ microglial cells in hNSCs-treated rats. **a** Representative confocal images of activated microglial cells (Iba1 + CD68+) in the anterior horn (AH) of CTRL, HBSS or hNSCs rats. **b** Chart showing the quantification analysis of the number of CD68+ cells in the anterior horn, anterior funiculus or whole (anterior horn, anterior funiculus and lateral funiculus). Scale bars: 75 μm

To assess whether hNSCs can influence resident inflammatory cells, we evaluated the morphology and quantity of GFAP+ cells (for astrogliosis, Fig. 5) and of Iba1+ and CD68+ cells (for microgliosis, Figs. 6, 7), in the L3–L4 segments of the spinal cord around the grafted area, and looked for how the activation correlated to disease progression (at 15, 40DAT and E-ST). To determine the magnitude of the effect that hNSCs have on the different components of the motor system, we separately evaluated the anterior horns (which contain the somata of α-MNs) and the white matter, specifically the anterior and lateral funiculi (which comprise the descending fibres of the pyramidal and extrapyramidal tracts, respectively). These areas, colonised by transplanted hNSCs (Fig. 1c–e), are directly involved in the neuronal circuitry for motor control and majorly affected by the inherent degeneration process in this animal model of ALS^12^.

#### Astrogliosis

At 15DAT, in both control groups, almost all the astrocytes in the anterior horns were in a reactive state, displaying hypertrophic and thick processes strongly immunopositive for GFAP (Fig. 5a, arrows). At 40DAT and at E-ST, astrogliosis was further enhanced (Fig. 5a, arrowheads). In contrast, in hNSC-transplanted SOD1 rats, the majority of astrocytes displayed thinner processes and a smaller soma. These morphological differences between animal groups were more evident at 40DAT (Fig. 5a; Supplementary Fig. 2A). A similar immunomodulatory effect of transplanted hNSC was also observed in white matter regions (Supplementary Fig. 2B). The quantitative analysis mirrored the morphological observations (Fig. 5b) by demonstrating, as early as 15DAT, a statistically relevant reduction of the number of astrocytes in hNSCs-transplanted SOD1 rats relative to the HBSS-treated group, both in the anterior horns and funiculi; this difference became more striking at 40DAT (*p* < 0.01 versus untreated SOD1 and *p* < 0.05 versus HBSS-treated SOD1). Finally, at E-ST, the hNSC-transplanted rats still showed a lower level of astrogliosis in the white matter tracts (Fig. 5b, anterior funiculi and whole, *p* < 0.05 versus the same in untreated SOD1 rats). To note, hNSC-treated animals reached E-ST at a significantly later age (mean age, P204) compared with control animals (mean age P170).

Astrocyte fractions were comparable at the mid-to-late stage transition (15 to 40DAT) in most animals that belonged to the same treatment group, but had decreased at E-ST, likely due to generalised cell damage that also involved astrocytes^32–34^.

#### Microgliosis

The immunomodulatory effect of hNSCs on the microglial component was mostly marked at 40DAT. In fact, we observed that at 15DAT, Iba1 + microglial cells displayed thick, rough prolongations (Fig. 6a, arrows) and were comparable among all groups. This inflammatory process was consistently enhanced within the 15 to 40DAT transition in the spinal cord of control groups. At these time points, Iba1+ cells were hypertrophic, with short processes and an “amoeboid” (activated) morphology (Fig. 6a, arrowheads; Supplementary Fig. 3A). In contrast, at 40DAT, in the anterior horns of hNSCs treated SOD1 rats, the switch of microglial cells to an enhanced activated state, appeared arrested (Fig. 6a; Supplementary Fig. 3A). No difference was observed between the groups at the E-ST (Fig. 6a). A similar pattern was also observed in white matter regions (Supplementary Fig. 3B). The quantification analysis correlated with the morphological observations, showing the reduced numbers of Iba1+ cells in hNSC-transplanted SOD1 rats at 40DAT (from *p* ≤ 0.05 to *p* ≤ 0.01 versus controls, Fig. 6b).

To evaluate the contribute of activated microglial/macrophages cells, we analysed the expression of CD68. Consistent with the above findings, CD68+ microglial cells displayed a morphologically activated state in the spinal cord of each experimental group (Fig. 7a; Supplementary Fig. 4A), and the CD68 protein co-localised with the pan-microglial antigen Iba1 (Supplementary Fig. 4B). The quantitative analysis confirmed that, at 40DAT, the number of activated CD68+ cells was reduced in hNSC-treated animals (*p* < 0.05 in the anterior horn, Fig. 7b).

## Discussion

ALS is a multifactorial disease with no available effective cure^1,2^. We present compelling evidence that intraspinal delivery of hNSCs ameliorates the course of the disease, delays the deterioration of motor functions and extends overall survival in a transgenic rat model of ALS. These favourable symptomatic outcomes (that peaked between 40–60DAT) are accompanied by a delay in the accumulation of histopathological markers of ALS, referable to a hNSCs pleiotropic mode of action^35–38^. In particular, anterior horn MNs are preserved and astrogliosis and microgliosis are reduced in the spinal cord tract of hNSC-treated SOD1 rats.

### Relevance to clinical studies

The positive effects of hNSCs transplantation on disease progression in models of ALS have been widely documented^11–15,22,39,40^, but the variability in the methodologies used to derive and expand hNSCs has often confounded interpretations. Our data are remarkably relevant because we used hNSCs derived with the AIFA-authorised protocol (aM 154/2018) that has been used for the ALS Phase I trial (EudraCT:2009–014484–39 trial)^23,26,41^. The rigorous experimental conditions that we describe in this study mimic the clinical setting with the goal of having our results be relevant to future phase II studies in patients.

In preclinical studies, often stem cells are delivered at the pre-symptomatic stage of the disease, in order to maximise the window during which the cells can exert their therapeutic actions^12,15,40^. But while this approach is optimal for obtaining positive results in a basic research setting, translating this therapeutic sequence—which involves making transplantations prior to the appearance of symptoms—to the bedside is clearly not realistic, because ALS diagnosis only occurs at the symptomatic stage. We thus chose to treat the animals when the initial motor symptoms of the pathology are recognisable. But despite the treatment being administered after disease onset and a corresponding reduction of the therapeutic window, hNSC-treated rats showed better motor performances compared with controls.

Because intraspinal cord injection partially disrupts the parenchymal structure and triggers the inflammatory reaction, worsening the animal’s motor functions^42,43^, we also compared the symptomatic progression of SOD1 rats treated with hNSCs to untreated SOD1 rats (CRTL)—the goal here was to control for possible detrimental effects of the injection procedure. As expected, the motor performances of treated animals were reduced compared with CTRL, in the immediate post-injection period. Notwithstanding, as early as 50DAT, hNSC-transplanted rats completely overcame the surgery-induced impairment, displaying a significantly improved motor performance relative to both control groups. Under our stringent experimental conditions, a symptomatic improvement relative to untreated animals has never been documented, further strengthening the clinical relevance of our approach and supporting our surgical procedure.

### Symptomatic improvement

Intra-spinal delivery is aimed at preventing the degeneration of secondary MNs that leads to paralysis, and ultimately to respiratory failure. The major limitation of this strategy is that therapeutic hNSCs are supplied to a restricted area of the motor control system, while ALS affects the entire motor neuraxis, including upper MNs in the cortex. Notwithstanding this, the survival of hNSC-treated rats was extended by ~30 days relative to control animals. Similar studies have reported a smaller increase in lifespan (between 11 and 17 days^14,15^ or no effect on survival^12^).

### Extensive migration and integration and no rejection

hNSCs migrated radially, from the anterior horn into the lateral and anterior funiculi of the white matter (comprising descending extrapyramidal and pyramidal fibres) as well as along the rostro-caudal axis, reaching the thoracic and sacral segments. The observation that different hNSC lines display an intrinsic variability in migration (unpublished data), suggests the necessity of an accurate evaluation of this parameter in preclinical phases, in order to improve the therapeutic efficacy of these stem cells in phase II trials.

Moreover, under conditions of transient immunosuppression, hNSCs are able to survive in SOD1 rats, up to the ESS, with no rejection and no adverse immunological effects due to cross-species differences, consistently with our previous studies^44^. Our findings validate the wide therapeutic potential of hNSC in that they show virtually zero immunogenicity. This aspect, of course, must be further verified with regard to the absence of rejection after the cells have been transplanted.

### The slow-down of symptomatic worsening corresponds to an amelioration of the pathogenic environment including neuroinflammation blunting by hNSCs

Here we present, for the first time, a time course analysis to evaluate the progression of astrogliosis and microgliosis in order to reinforce the notion that the inflammatory cascade is an efficacious therapeutic target for the transplanted hNSCs^44–46^. Our analysis points to a correspondence between the delay in worsening of symptoms observed in the hNSCs-treated SOD1 rats and the reduced inflammatory response in the anterior horns. In fact, at 15DAT, the three experimental groups showed minimal differences, concerning both neuroinflammation and behavioural symptoms. While at 40DAT, activated astroglial and microglial cells were significantly reduced in hNSCs-treated rats, at the morphological level as well as by quantitative evaluation. These differences were accompanied by evidence of the first symptomatic benefits (motor score at 40DAT), which continued through the subsequent evaluation period (40–60DAT).

Within the same time frame (40DAT), the misfolded SOD1 deposits were less present in the anterior horns of hNSCs-transplanted rats respect to both control groups. Although preliminary, this observation is consistent with the hypothesis that hNSCs ameliorate the pathogenic surroundings of MNs, in fact it is now well established that misfolded SOD1 accumulation is a key element of SOD1-mediated ALS, and that the reduction of its level determines significant beneficial effects in preclinical model^47,48^.

Hefferan et al.^12^ have demonstrated that axons of descending primary MNs in the SOD1 rat model are impaired. Notably, these degenerative processes are more evident in the lateral (mostly rubrospinal tract) and anterior (pyramidal pathway) white matter. In addition, when the corticospinal tract is damaged, lateral columns provide de novo innervating cells to MNs in the anterior horns^49,50^, suggesting that lateral columns contribute to recovery mechanisms following degenerative insults to the motor system. Considering the extensive migration of hNSCs from the anterior horns to the aforementioned areas (lateral and anterior white matter), we evaluated and quantified a significant reduction of neuroinflammation in all these areas that were maintained until the late stages of the disease, with an obvious amelioration of the local pathogenic environment. Given the reports of axon dieback (i.e., axonal degeneration might occur prior to the onset of symptoms) in the pathogenesis of ALS^51–53^, the amelioration of parenchymal milieu surrounding distal axons—such as the reduction of neuroinflammation—could retrogradely affect the MN soma located in the motor cortex. Thus, it probably contributes to the generalised beneficial effect of hNSCs, in spite of their local delivery.

In conclusion, our study points to the potential therapeutic efficacy of using clinical-grade hNSCs that are already in use in a clinical trial on ALS patients. Our findings validate that extensive and non-invasive intraparenchymal transplantation of hNSC lines, which have been cultured under standardised conditions, can work as a therapy for ALS.

## Materials and methods

### Animal model—Ethics Statement and Institutional Animal Care and Use Committee (IACUC) approvals

SOD1 transgenic male rats (Taconic, USA) were bred with Sprague Dawley female rats (Harlan Laboratories, USA) to obtain and maintain hemizygous transgenic progeny—the transgenic offspring were identified by polymerase chain reaction (PCR), in which DNA extracted from the tails at P22 was amplified and used to verify the presence of the exogenous human SOD1 gene^28^. All animal care and experimental procedures were carried out according to the current national and international animal ethics guidelines, and were approved by the Italian Ministry of Health (authorisation number # 286/2013 -B).

### Preparing hNSCs for implantation

The hNSCs used in the study have been produced and characterised in the Cell Factory and Biobank of Santa Maria Hospital (Terni, Italy), authorised by the Italian Medicine Agency (AIFA) for the production of hNSCs to be used for clinical trials (aM 54/2018). The methodology applied to isolate, expand, characterise and cryopreserve the lines is based on the Neurosphere Assay^26,41,54^, and has been used for the production of the cells utilised in phase I trials for Amyotrophic Lateral Sclerosis patients (NCT01640067^23^) and for Secondary Progressive Multiple Sclerosis patients (NCT03282760, ongoing).

The entire production process, starting from tissue procurement to cryopreservation is compliant to cGMP guidelines and approved by AIFA. The hNSCs are obtained from foetal brain tissue derived from fetuses that underwent miscarriage or natural in utero death upon receiving the signed informed consent from the mother. Forty-eight hours prior to implantation, hNSCs were plated in the growth medium at a concentration of 10,000 cells/cm^2^. On the day of surgery, hNSCs were collected by centrifugation, viable cells were counted by Trypan blue exclusion criteria, and the correct number of cells were re-suspended in HBSS for the transplant.

### Experimental groups

SOD1 transgenic male rats were randomly divided into three experimental groups: (i) transplanted with hNSCs (hNSC rats, *n* = 15); (ii) treated with HBSS (HBSS rats, *n* = 15) and (iii) untreated (CTRL rats, *n* = 22). An additional group of non-transgenic littermates (wild-type, WT, *n* = 9) were used as controls for symptomatic evaluation of the colony. Tacrolimus (FK506) and cyclosporine (cyclosporin A) are the principal immunosuppressive drugs that have been applied for solid organ transplantation^55,56^ and have been translated to stem cell treatments for PD^57^ and ALS^22^. In animal models, despite differences in potency, both drugs showed excellent survival rates for grafts across many comparative studies^58,59^. Our previous results^44,45^ showed that hNSCs can survive—without signs of rejection—in the rat brain up to 6 months under transient immunosuppression treatment, with cyclosporin A. On the bases of these results, we applied the same immunosuppressive treatment with administration of cyclosporine A (15 mg/kg/day subcutaneous; Sandimmne, Novartis) that was initiated on the day of transplantation and continued for 15 days after surgery (for all animal groups).

### Implanting hNSCs into spinal cord

Cells were transplanted at the early-symptomatic stage (ESS) of the disease, i.e., when the SOD1 rats presented with initial signs of a decrease in limb control and/or with initial failure on the rotarod. Animals were anaesthetised with isofluorane (1.5–2% maintenance; in room air), placed in a spinal clamp apparatus (David Kopf Instruments, Tujunga, CA), and a partial L1–L2 laminectomy was performed. The cells (or HBSS) were injected bilaterally with use of a Hamilton syringe (33-gauge needle) into the ventral horns of the L3–L4 segments of the cord. Injections were made on both sides, at four sites, set 1 mm apart. At each site, we delivered 1 μl of HBSS containing the hNSCs (100,000 cells/μl), or 1 μl of HBSS only (in control group), applied over 60 s; the needle was left in place for an additional 180 s, then withdrawn. After all injections were completed, the incision was sutured and animals were placed back into their cage with ad libitum access to food and water.

### Assessment of neurological function and disease progression

Motor functions were evaluated using three different tests: rotarod, motor score and body weight^27,28^— these tests allowed us to define the ESS, the MSS, the LSS and the E-ST of the disease (see details in the Results section). All behavioural measures were recorded by individuals blinded to the treatment group.

#### Rotarod test

The rotarod test was used to evaluate general motor coordination, and the strength and balance of the animal. Starting on P70, animals were trained on the rotating rod, increasing the sped from 4 to 22 rotations per min within 300 s; the rats were trained three times a week, and then tested twice a week^27^). Each test consisted of three runs on the rotarod (lasting a maximum of 300 s) and performance was quantified by calculating the mean time (seconds) spent on the rod.

#### Motor score

Rats were scored twice a week, starting on P70. Hind-limb splay defects were scored on a scale of 0–4, with 4 = normal; 3.5 = onset of hind-limb splay defect; 3 = abnormal gait; 2 = partial hind-limb paralysis (first signs of dragging); 1 = hind-limb paralysis plus forelimb weakness; 0 = significant fore- as well as hind- limb paralysis.

#### Body weight evaluation

Rats were weighed twice a week, starting on p70.

#### Statistical evaluation of symptomatic analysis

Temporal evolution of rotarod performance, motor score and body weight were assessed using a longitudinal linear model, accounting for the repeated, unequally spaced, measurements design using a spatial power covariance matrix. Nonlinear evolutions were modelled by adding both linear and time quadratic terms into the model. Pairwise group comparisons (CTRL vs. HBSS vs. hNSCs) of overall and specific time points were assessed with use of suitable statistical contrasts. Estimated means, and their temporal evolution, were graphically represented. Time to death analysis was conducted with use of a proportional hazard Cox regression model, and risks were reported as hazard ratios (HR), along with their 95% confidence intervals (95% CI). The time variable was defined as the time between the surgery (or, for CTRL rats, the time of initial deterioration of symptoms) and the date of death. Kaplan–Meier curves were also displayed. A *p*-value of < 0.05 was considered to be statistically significant. All analyses were performed using SAS Release 9.3 (SAS Institute, Cary, NC). Note that we performed a sensitivity analysis by choosing a CTRL group that presented, on day 0, a rotarod and motor score value that was better than that for the hNSC and HBSS animals (*p* < 0.05).

### Immunohistochemistry

Histological analyses were performed at three time points: 15DAT, 40DAT and END STAGE. To the scope, we killed *n* = 5 animals/time point out of the previously described experimental group (transplanted with hNSCs, treated with HBSS and untreated). To retrieve the spinal cord, animals were euthanized by overdose of anaesthesia and perfused/fixed with paraformaldehyde solution (4%). Immunohistochemistry was performed on cryopreserved coronal sections of the spinal cord fixed in 4% PFA made up in PBS. Cords were removed, immersed overnight in 4% PFA at 4 °C, cryoprotected in sucrose gradients (10, 20 and 30%) and sectioned at 30 μm on a cryostat. Parallel series were collected on glass slides. For immunohistochemistry, sections were incubated for 90 min with blocking solution (PBS-1X with 10% normal goat serum (NGS) and TRITON X-100 at 0.1%) at room temperature, then immersed overnight in the following primary antibodies, diluted in the blocking solution, at 4 °C: human nuclei (huN mAb, Millipore), glial fibrillary acidic protein (GFAP pAb, Dako), Ki67 antigen nuclear (Ki67 pAb, Monosan), Ionised calcium-binding adapter molecule 1 (Iba1 pAb, Wako), cluster of differentiation 68 (CD68, mAb, Biorad), doublecortin (DCX, pAb, Santa Crouz), platlet-derived growth factor receptor-α (PDGFR-α, pAb, Euroclone), human Nestin (hNestin, mAb, R&D system), β-tubulin III (β-tubIII, mAb, Covance), anti SOD1 exposed dimer interface (SEDI, pAb StressMarq Bioscineces Inc., SPC-206P, generously provided by Corona C. and Crociara P.). Tissues were rinsed with PBS. Samples were then incubated for 90 min at room temperature, in the following secondary antibodies: Alexa Fluor 594- or 488-labelled anti-mouse and/or anti-rabbit (Molecular Probes). After washing with PBS, the cell nuclei were labelled with DAPI for 20 min at room temperature. Finally, the slides were rinsed in PBS, coverslipped, and left to dry at room temperature in the dark. Microphotographs were acquired using a Zeiss Axiovert 200 direct epifluorescence microscope (Axioplan 2, Carl Zeiss, Jena, Germany) or by confocal microscopy (Leica DM IRE2 or Nikon).

### Stereological counts of MNs

One set of serial sections (every 1050 μm) from each animal was Nissl-stained for stereological counting. Nucleoli of MNs in the L1–L4 spinal cord (where cells were delivered) were identified and counted at 40x in (hNSC rats, *n* = 5; HBSS rats, *n* = 5; CTRL rats, *n* = 5): only neurons ≥ 200 μm^2^ in area (attributable to alpha motor neurons) and located in a proper position (http://store.elsevier.com/The-Spinal-Cord/isbn-9780123742476) were included. A 3.5 -µm guard zone, a 150 -µm × 150 -µm counting frame size and a 200 -µm × 200 -µm scan grid size were set. The density of MNs (cells/mm^3^) was calculated by counting cells with the Optical Fractionator stereological technique^60^, on a computer-assisted a Nikon Eclipse E600 microscope equipped with the StereoInvestigator software (MicroBrightField, Williston, VT, USA). Cells were counted on the computer screen, with the help of an Optronics MicroFire digital camera.

### Quantitative analysis of immunohistochemistry

Surviving transplanted hNSCs were determined by counting all huN + cells in serial sections through the spinal cord sections that spanned the graft area. The total number of hNSCs was calculated for the entire graft region, using the Abercombie formula. Data were presented as the percentage of surviving cells over the total number of transplanted cells. The full extent of anteroposterior migration was calculated by evaluating the distance between the most proximal and most caudal sections containing huN + cells. Differentiation of hNSCs was assessed by co-localisation of huN reactivity with markers depicting differentiation of the neural lineage: Ki67, Nestin, GFAP, DCX, β-Tub III, PDGFR-a. Analysis was performed on at least three sections within the transplant area, using three fields (×40 magnification) for each section.

To determine the numbers of active astroglia and microglia, we counted cells positive for GFAP, Iba1 or CD68 and divided by the total number of DAPI-stained nuclei. Data were quantified on bilateral epifluorescence images of the anterior horns, anterior funiculi and lateral funiculi acquired on the camera from at least three sections within the transplanted area (L3–L4 region).

Statistical analysis was performed by one-way ANOVA. Data were reported as means ± SEM. Each value represented the average of *n* = 5, animals, unless stated otherwise. Data were not considered statistically significant unless indicated in the figures (*p < 0.05, **p < 0.01, ***p < 0.001).

## Supplementary information

Suppl. Fig. 1

Suppl. Fig. 2

Suppl. Fig. 3

Suppl. Fig. 4

Supplementary figure legends
