# Supplementary figures and images for: Transplantation of clinical-grade human neural stem cells reduces neuroinflammation, prolongs survival and delays disease progression in the SOD1 rats
Source: Cell Death Dis. 2019 Apr 25;10(5):345. doi: 10.1038/s41419-019-1582-5 (PMC6484011; doi:10.1038/s41419-019-1582-5)

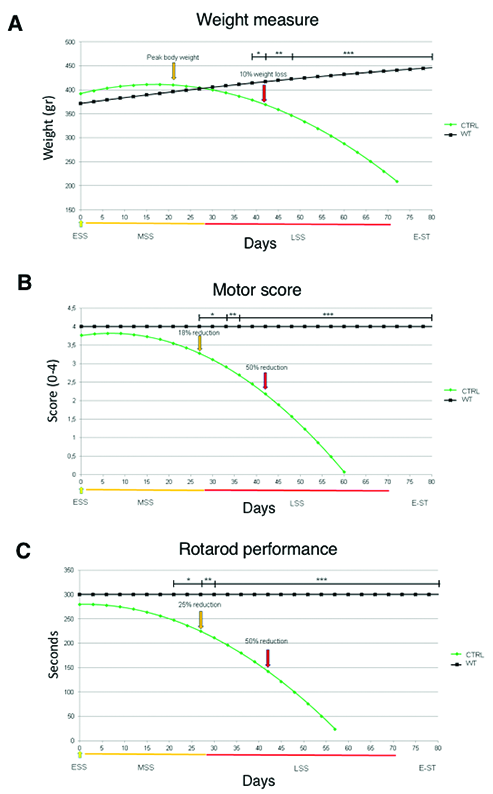

Supplement: Supplementary file 1 — Suppl. Fig. 1 [file 41419_2019_1582_MOESM1_ESM.tif]

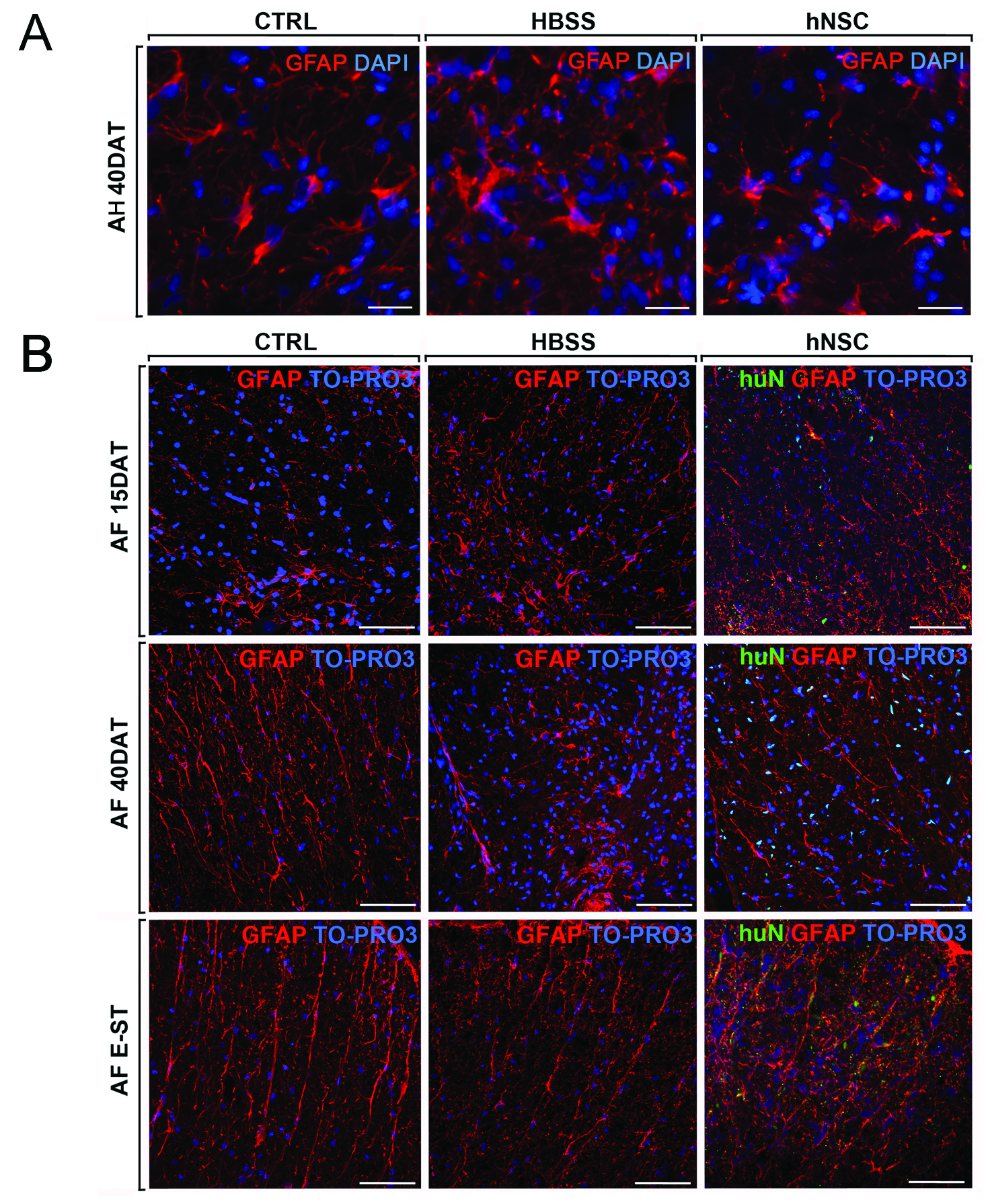

Supplement: Supplementary file 2 — Suppl. Fig. 2 [file 41419_2019_1582_MOESM2_ESM.tif]

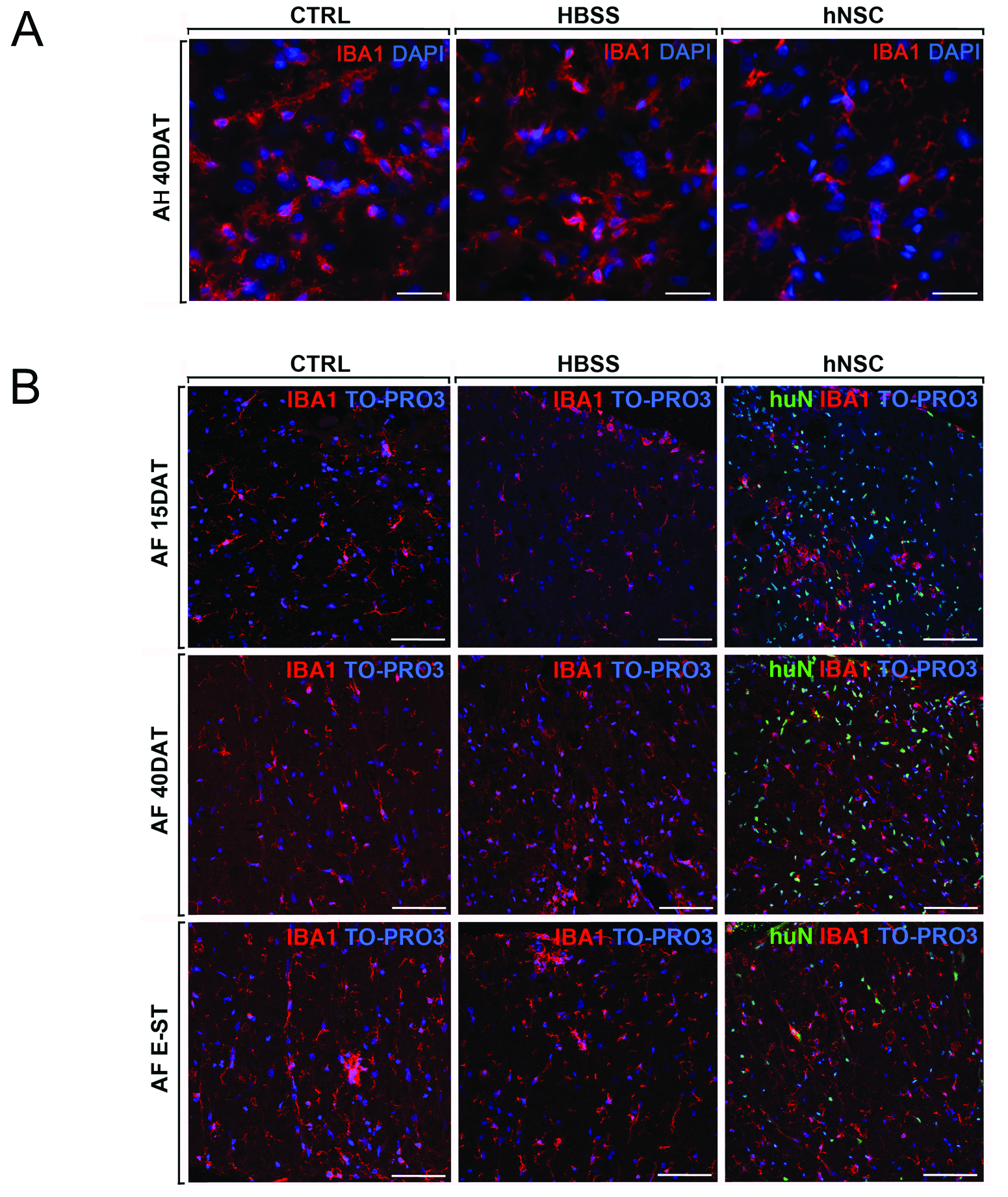

Supplement: Supplementary file 3 — Suppl. Fig. 3 [file 41419_2019_1582_MOESM3_ESM.tif]

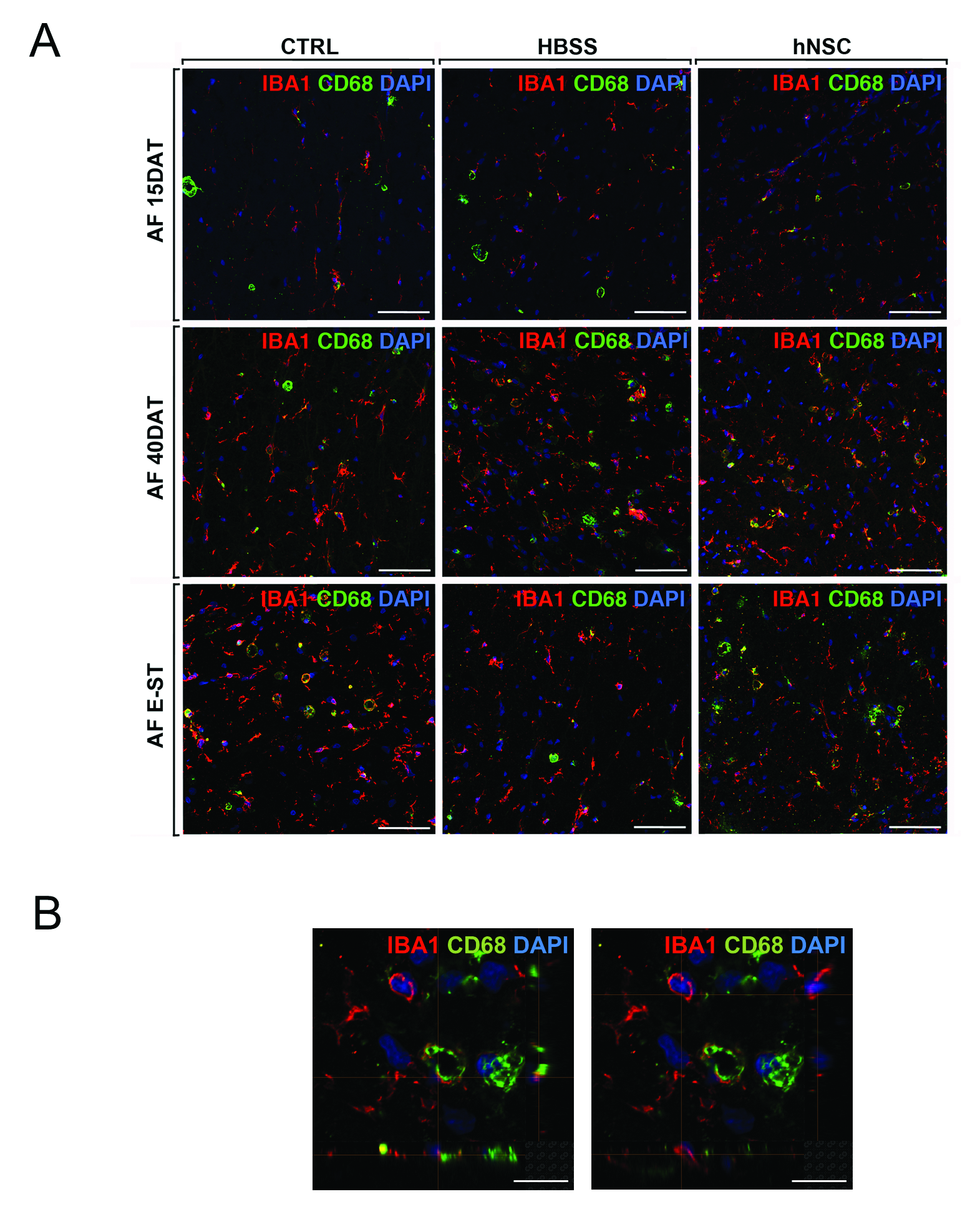

Supplement: Supplementary file 4 — Suppl. Fig. 4 [file 41419_2019_1582_MOESM4_ESM.tif]
