# Supplementary material for: Transplantation of clinical-grade human neural stem cells reduces neuroinflammation, prolongs survival and delays disease progression in the SOD1 rats
Source: Cell Death Dis. 2019 Apr 25;10(5):345. doi: 10.1038/s41419-019-1582-5 (PMC6484011; doi:10.1038/s41419-019-1582-5)
Supplement: Supplementary file 5 — Supplementary figure legends [file 41419_2019_1582_MOESM5_ESM.docx]

SUPPLEMENTARY FIGURE 1. Trend of weight, motor score and Rotarod performance decline in CTRL (green, n=22) or Wild Type (WT, black n=9) rats. The curves were obtained by applying a statistical modeling to the progressive reduction of the means values of weight, motor score and rotarod for each group. Statistical significance of the difference between the two groups is indicated with asterisks above the black bars. The symptomatic comparison between the two groups is shown starting from the ESS, considered as day 0. Colored bars below the x axe indicate the stages of the disease. *p≤0,05 ; **p≤0,01; ***p≤0,001. Data are reported as mean ± SEM.

SUPPLEMENTARY FIGURE 2. Panel (A) Representative images showing morphological differences between astroglial cells in the anterior horns of controls rats respect to transplanted ones. Scale bars: 25 μm. Panel (B): Representative confocal images of activated astroglial cells (GFAP+) in the anterior funicus (AF) of CTRL, HBSS or hNSCs rats. Scale bars: 75 μm

SUPPLEMENTARY FIGURE 3. Panel (A): Representative images showing morphological differences between microglial cells in the anterior horns of controls rats respect to transplanted ones. Scale bars: 25 μm. (B) Representative confocal images of activated microglial cells (Iba1+) in the anterior funiculus (AF) of CTRL, HBSS or hNSCs rats. Scale bars: 75 μm

SUPPLEMENTARY FIGURE 4. Panel (A): Representative confocal images of activated microglial cells (Iba1+CD68+) in the anterior funiculus (AF) of CTRL, HBSS or hNSCs rats. (B) Colocalization between Iba1+ and CD68+. Scale bars: 75 μm in A and 10 μm in (B).
